# Cogni-e-SpinDB 1.0: Open Dataset of Electrospinning Parameter Configurations and Resultant Nanofiber Morphologies

**DOI:** 10.1038/s41597-025-06520-5

**Published:** 2026-01-03

**Authors:** Mehrab Mahdian, Tamas Stummer, Norman Sepsik, Ferenc Ender, Diana Balogh-Weiser, Tamas Pardy

**Affiliations:** 1 Thomas Johann Seebeck Department of Electronics, Tallinn University of Technology, Tallinn, Estonia; 2 Department of Organic Chemistry and Technology, Budapest University of Technology and Economics, Budapest, Hungary; 3 Department of Electron Devices, Budapest University of Technology and Economics, Budapest, Hungary

**Keywords:** Nanoscience and technology, Materials science, Engineering, Polymer chemistry

## Abstract

Electrospinning is a versatile technique for producing nanofibers by elongating and depositing a polymer solution in an electrostatic field. Nanofiber quality is governed by process, environmental, and solution parameters, requiring extensive fine-tuning. Optimization is largely driven by independent experimental data, yet few openly available datasets exist to support modeling or provide reference parameters for stable nanofiber formation. We present Cogni-e-Spin DB 1.0, a dataset containing 809 experimental records of electrospinning parameters and corresponding nanofiber morphologies. This is the first large-scale, diverse, and machine-learning-ready dataset designed to address data scarcity. Its scale and diversity enable comprehensive process-structure-property analyses and support machine learning methods to gain insights into the complex electrospinning process. To assess the dataset’s reliability and utility, we verified data integrity against source publications and trained machine learning models as a proof of concept. These data are intended to accelerate fundamental research, model training, and process optimization across various electrospinning applications. We also developed a companion web platform to enable live dataset contributions and interactive exploration, fostering continuous community-driven updates.

## Background & Summary

Electrospinning, compared to other methods of nanofiber membrane formation, has the advantage of simple instrumentation, wide material selection, good efficiency and potential of scalability^1,2^. However, process control is challenging due to the complex interaction between process, environmental and solution parameters^3,4^. This is particularly relevant for multi-material, multi-layer membrane formation, which is essential in biomedical applications^5,6^. Experimentally determining the working ranges is time-consuming, whereas simple closed-loop control approaches that work well for water as a solvent may not readily extend to more volatile solvents^7^. Given these challenges, there is a clear need for a tool that helps to comprehensively understand the electrospinning parameter space and model the effects of experimental parameters on nanofiber product morphology.

The input parameter space of electrospinning can be sub-divided into solution (e.g. concentration, molecular weight, and conductivity), process (e.g. applied voltage, feed rate, tip-collector distance), and environmental parameters (e.g. temperature, humidity)^4,8,9^. On the output side, nanofiber morphology is typically quantified by fiber diameter, which affects product performance and applicability. For instance, diameter affects surface area and porosity, which in turn affect permeability in filtration applications and diffusion rate in membranes^6,10^. From a systems and/or process engineering perspective, modeling the relationship between input parameters (solution, environmental, process) and nanofiber product morphology is essential. Given the complexity of the electrospinning parameter space, machine learning provides a promising approach for modeling input-output relationships. However, this needs adequate amounts of high-quality data of confirmed parameter input to product morphology output relationships for model-building.

In the past, analyses presenting numerical records from experiments focused primarily on solution properties and their relation to nanofiber product morphology and applicability^4,11–15^. Process parameters have been covered less frequently, and environmental parameters the least. Data in these works have been typically reported in tables in the main text and would need to be extracted for modeling purposes. In addition, works presenting data from several experiments have focused on subsets of the total parameter space relevant to the scope of the application being analyzed^11,16^.

The recent surge in machine learning (ML) applications for electrospinning process modeling underscores this critical data need^17–19^. While these and other ML studies demonstrate the potential of data-driven approaches, they are inherently limited by the availability, scope, and ready-to-use integration of the underlying experimental data.

Existing open datasets, such as those focusing on specific material systems like PVDF in the FEAD database^20^, are valuable but often limited in material diversity. This dataset, comprising of 745 records, focuses on one type of polymer system (PVDF) with different solvent system, derived from 52 studies. This study represents an important initial effort, however, its utility is constrained by its narrow scope, focusing exclusively on a single polymer, and its limited final size of approximately 340 curated records. Furthermore, its original data structure was not optimized for direct computational use.

Other modeling efforts often rely on fragmented data extracted from the literature, which lacks standardization. This gap highlights the need for a dedicated, large-scale, and machine-learning-ready data resource. Therefore, with the Cogni-e-Spin DB 1.0, we aimed to collect the largest possible set of electrospinning experimental records composed of input parameters and the resulting nanofiber morphology, and formatted it in a machine learning-friendly manner.We believe that this dataset will provide a solid starting point for training machine learning models for predicting electrospinning parameter configurations that result in stable nanofiber formation. Our core contribution is the creation and provision of a large-scale, curated dataset that is formatted for immediate use in machine learning pipelines. Assembled from 57 sources; it is the first dataset of its scale, encompassing a diverse set of 12 materials and 809 complete records, and is structured according to modern data science standards (including standardized variable names adhering to PEP8 guidelines) to ensure ease of use and reproducibility.

The dataset supports supervised learning methods, including regression models, which can predict quantitative outcomes (e.g., fiber diameter) and classify qualitative results (e.g., fiber formation stability). These approaches also facilitate Process-Structure-Property analysis and anomaly detection. Moreover, integrating techniques such as eXplainable AI (XAI) allows interpretation of parameter importance and provides actionable insights for process optimization. Successful application requires sufficient high-quality data linking input parameters to observed nanofiber outcomes. We believe this dataset provides a foundational resource that will facilitate more robust, generalizable, and comprehensive machine learning models for predicting electrospinning outcomes, thereby accelerating research and process optimization in the field.

## Methods

**Fig. 1:**
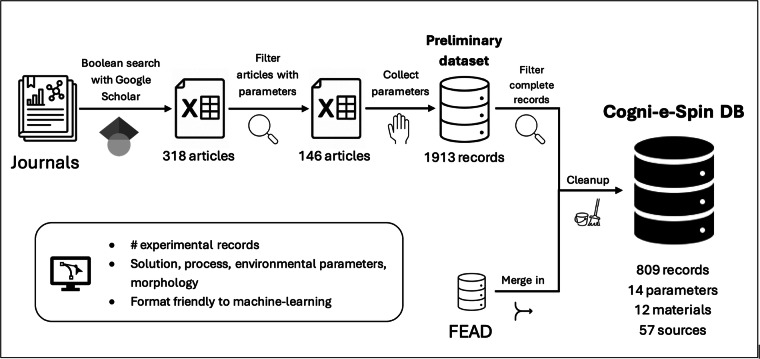
The general scheme of Cogni-e-Spin DB 1.0 construction.

In this research, we compiled Cogni-e-Spin DB 1.0^21^ from data in peer-reviewed publications, including any pre-existing open dataset containing experimental records of electrospinning (Fig. 1).

First, we conducted a parametric search of peer-reviewed journal and conference papers using Google Scholar with Boolean search terms, as detailed in the -“Search Terms” section of our GitHub README (link under Code Availability). We combined multiple Boolean queries to maximize the number of relevant peer-reviewed papers. This initial search yielded 318 entries, of which about two-thirds were technical reports. The list also included some non-scientific materials (e.g. industrial product brochures on electrospinning), which were kept as informational items but disregarded in later processing. The full, unfiltered list is available in the Excel worksheet titled ‘Table 1’ in the preliminary dataset hosted on TalTech Data Repository^22^.

Next, we manually screened these reports to include only those with clearly reported quantitative data, primarily found in tables, images or in the text. Reports that contained only qualitative analyses were excluded. Critically, regardless of the document type (review, modeling, or experimental report), only primary, quantitative experimental data, representing a confirmed parameter input to product morphology output relationship was extracted. After this filtering, 146 distinct sources remained.

From these sources, we extracted relevant parameters and compiled a preliminary dataset, available in the Excel worksheet titled ‘Table 2’ in the preliminary dataset hosted on TalTech Data Repository^22^. This preliminary dataset contained 1913 records, though some records had missing parameters (marked as “N/A” in the Excel).

In the next step, we filtered the preliminary dataset for complete records and merged in pre-existing datasets, such as FEAD^20^, to ensure consistency and completeness. Then we cleaned up the dataset according to the following principles:

- Unified polymer and solvent designations to eliminate ambiguity. For example, “Acrylonitrile polymer” and “Polyacrylonitrile” were both standardized to “PAN.” This ensured consistent naming conventions across the dataset.
- Standardized variable names per PEP8 guidelines to enhance code readability and reproducibility.
- Eliminated duplicates made during dataset compilation, some entries were found to be duplicates, either identical data reported in multiple articles or repeated within the same source. For these, we identified the original articles and manually screened the data to remove duplicates. Typically, if two entries had the same data but different sources, the older publication’s value was retained, while the newer one was discarded. This process was only applied to perfectly identical records, preserving distinct measurements from different publications.
- Converted units to create a uniform dataset. Fields reported in different units, where possible, were converted into the following standardized units across all records. This harmonization improved consistency and comparability.
  - Flow rate: ml/h
  - Needle diameter: G (Gauge)
  - Concentration: wt% or w/v% (as originally reported, but with unified naming)
  - Voltage: kV
  - Tip–collector distance: cm
  - Temperature: ∘C
  - Humidity: %
  - Fiber diameter: nm
  - Fiber diameter variation: nm
  - Rotation speed: RPM
- Normalized fiber diameter variations reported as standard deviations or percentages into a consistent format to ensure comparability across datasets.
- Constructed JSON data structure to dynamically represent complex material and solvent compositions and process setups. This standardized format facilitates flexible and consistent data handling.
- Dropped records for precursor polymer materials with fewer than 10 observations. This cutoff aligns with widely accepted sample size heuristics, as very small groups tend to produce unstable parameter estimates and increase the risk of overfitting. For instance, Peduzzi *et al*.^23^ demonstrated that models with fewer than 10 events per predictor variable produce biased coefficients and unreliable inference, a principle that applies broadly to regression modeling. Similarly, Figueroa *et al*. showed that small sample sizes substantially degrade predictive performance in supervised learning tasks^24^.
- Dropped records with solvents that had fewer than 5 entries
- Flagged tail observations in the nanofiber diameter for each precursor polymer material. These represent extreme data points that deviate substantially from the typical distribution of diameters. However, such points are not necessarily invalid; thus, we refer to them as ‘tail observations’ rather than ‘outliers,’ acknowledging natural variability without excluding these data. Tail observations were identified using the interquartile range (IQR) method, where values below *Q*_1_ − 1.5 × IQR or above *Q*_3_ + 1.5 × IQR were flagged. This method was applied within each polymer group, and the results were recorded as a boolean field (tail_observation) in the dataset to facilitate flexible filtering and ensure reproducibility.

With this, we finalized the Cogni-e-Spin DB 1.0^21^ dataset, which included 809 records for 14 parameters, 12 materials, and 57 sources^25–81^.

## Data Records

Cogni-e-Spin DB 1.0 can be accessed online within Zenodo^21^. The main dataset is structured as a downloadable CSV format data record. A description of each metadata field is provided in Table 1. The dataset comprises 20 distinct fields, covering four core categories of information essential for electrospinning analysis: source attribution (DOI), material composition (polymer(s), solvent(s), concentration), process parameters (voltage, flow rate, tip-collector distance, needle diameter, collector type), and nanofiber outcomes (fiber diameter, diameter variation, formation stability, and tail observation flags).

**Table 1:** Data structure of electrospinning experimental records with detailed descriptions.

Column	Description	Type
doi	Digital Object Identifier in URL format corresponding to the paper	string
polymer(s)	Polymer name described by acronym(s)	string
is_solvent_blend	Indicates if the solvent is a blend (True/False)	boolean
solvent(s)	Solvent name used	string
solvent_components	Detailed composition of solvent components	JSON object
solution_concentration	Concentration of the solution	float
solution_concentration_unit	unit of solution concentration [wt% and w/v%]	string
needle_type	Type of needle used in electrospinning	string
needle_diameter_g	Diameter of the needle [G]	int
collector_type	Type of collector used	string
rotation_speed_rpm	Rotation speed of the collector in case of rolling drum [RPM]	int
voltage_kv	Applied voltage during electrospinning [kV]	float
flow_rate_ml/h	Flow rate of solution through needle [ml/h]	float
tip_collector_distance_cm	Distance between needle tip and collector [cm]	float
temperature_c	Ambient or controlled temperature during experiment [°C]	float
humidity_%	Ambient humidity level [%]	float
was_formation_stable	Indicates if fiber formation was stable (True/False)	boolean
fiber_diameter_nm	Average fiber diameter [nm]	float
fiber_diameter_variation_nm	Variation (standard deviation) in fiber diameter [nm]	float
tail_observation	Indicates presence of tail formation in fibers (True/False)	boolean

**Fig. 2:**
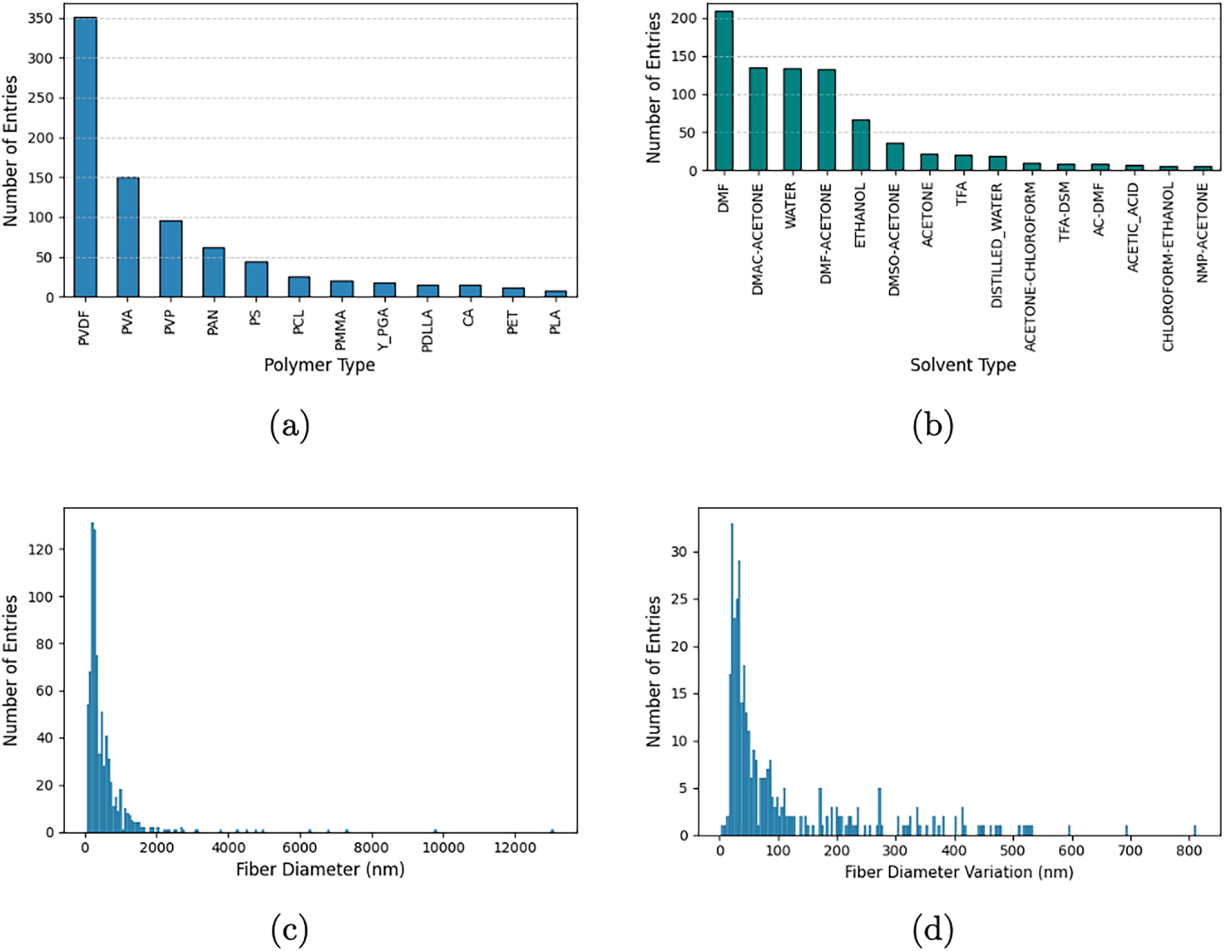
Statistics in Cogni-e-Spin DB 1.0. (**a**) Distribution of entries based on precursor polymers, (**b**) Distribution of entries based on solvents, (**c**) Distribution of entries based on product nanofiber diameter, (**d**) Distribution of entries based on product nanofiber diameter variation.

The dataset composition and key characteristics are summarized in Fig. 2. A wide range of precursor polymers is covered, with PVDF, PVP, PAN, and PVA being the most frequently reported, reflecting their broad applicability in electrospinning research (Fig. 2a). The solvent usage also shows considerable diversity, with both single and binary solvent systems present. DMF, and its mixtures with acetone and water, appear most frequently, underscoring their common role in dissolving a variety of polymers (Fig. 2b). In terms of morphological outcomes, the fiber diameter distribution (Fig. 2c) spans more than an order of magnitude, ranging from ultrafine nanofibers (~10nm) to thicker microfibers (≥10 *μ*m), with a sharp mode below 500nm and a long-tailed distribution extending into the micron scale. This wide range underscores the versatility of electrospinning parameters and material systems captured in the dataset. Moreover, (Fig. 2d) presents the distribution of fiber diameter variation (standard deviation), with the majority of entries concentrated below 200nm, enabling robust analyses of uniformity and process consistency. These statistics collectively demonstrate the broad scope, resolution, and relevance of the dataset for data-driven analysis and modeling in electrospinning research.

## Technical Validation

The dataset validation focused on two aspects: (1) verification of data integrity and (2) demonstration of utility for data-driven modeling.

All experimental data were originally published in peer-reviewed scientific journals. Therefore, potential errors in our dataset may stem only from mistakes during data extraction or summarization. Data extraction for the preliminary dataset benefited from the scale achieved by engaging multiple contributors. To ensure consistency across this work and to minimize transcription bias, the final curator performed a quality control cross-check, comparing selected records in the final dataset against their original sources, confirming consistency and accuracy.

To demonstrate the practical utility of Cogni-e-Spin DB 1.0, we conducted a machine learning experiment using a subset of the dataset filtered for stable nanofiber formation and flat collector setups, focusing on PVDF and PVA polymer systems, which together comprise the majority of the dataset, are widely used in electrospinning applications such as biomedical scaffolds and energy devices^82,83^, and reflect typical experimental practice where modeling is often performed on a single polymer system using curated or in-house data^84–86^.

We did not restrict modeling to specific solvent types, as the polymer-solvent combination is inherently reflected in the solution concentration. After filtering, 187 PVDF and 133 PVA records remained; however, 88 PVDF and 96 PVA entries lacked values for needle diameter. In the final published dataset, these records retain their missing value status (N/A) to maximize user flexibility. To enable fair comparison between modeling approaches, missing needle diameter values were imputed with the median value (18 G) calculated from the available data within each polymer subset.

We trained a simple linear regression for baseline and CatBoost regression model^87,88^ separately on each polymer subset to predict average fiber diameter using the following input parameters: solution concentration, applied voltage, flow rate, tip-to-collector distance, and needle diameter (where available). CatBoost is a gradient boosting framework well suited for small- to mid-sized structured datasets, offering regularization techniques that reduce overfitting.

To evaluate model performance, we employed 5-fold cross-validation. This strategy partitions the dataset into five subsets, iteratively using four for training and one for testing. Averaging performance across all folds mitigates variance and provides a robust estimate of generalization.

We used the following standard regression metrics:

- **Root Mean Squared Error (RMSE)**: penalizes larger errors.
  1 $$RMSE=\sqrt{\frac{1}{n}\mathop{\sum }\limits_{i=1}^{n}{({y}_{i}-{\widehat{y}}_{i})}^{2}}$$
- **Mean Absolute Error (MAE)**: average magnitude of prediction errors.
  2 $$MAE=\frac{1}{n}\mathop{\sum }\limits_{i=1}^{n}| {y}_{i}-{\widehat{y}}_{i}| $$
- **Coefficient of Determination** (*R*^2^): proportion of variance in the target variable explained by the model.
  3 $${R}^{2}=1-\frac{{\sum }_{i=1}^{n}{({y}_{i}-{\widehat{y}}_{i})}^{2}}{{\sum }_{i=1}^{n}{({y}_{i}-\bar{y})}^{2}}$$

For the PVDF subset, CatBoost achieved an *R*^2^ of 0.70 ± 0.20 and an MAE of 94.46 ± 20.75nm, compared to the Linear Regression baseline performance of *R*^2^ 0.12 ± 0.22 and MAE 266.49 ± 45.19nm. For the PVA subset, CatBoost resulted in an *R*^2^ of 0.43 ± 0.26 and an MAE of 40.73 ± 13.94nm, while Linear Regression showed an *R*^2^ of 0.43 ± 0.15 and an MAE of 62.27 ± 12.69nm.

The difference in R^2^ values between the two subsets (0.70 for PVDF vs 0.43 for PVA) reflects differences in the dataset characteristics. The PVA subset is smaller (133 records vs. 187 records for PVDF) and exhibits greater variability in reported input parameters, which is consistent with a more heterogeneous experimental space.

**Fig. 3:**
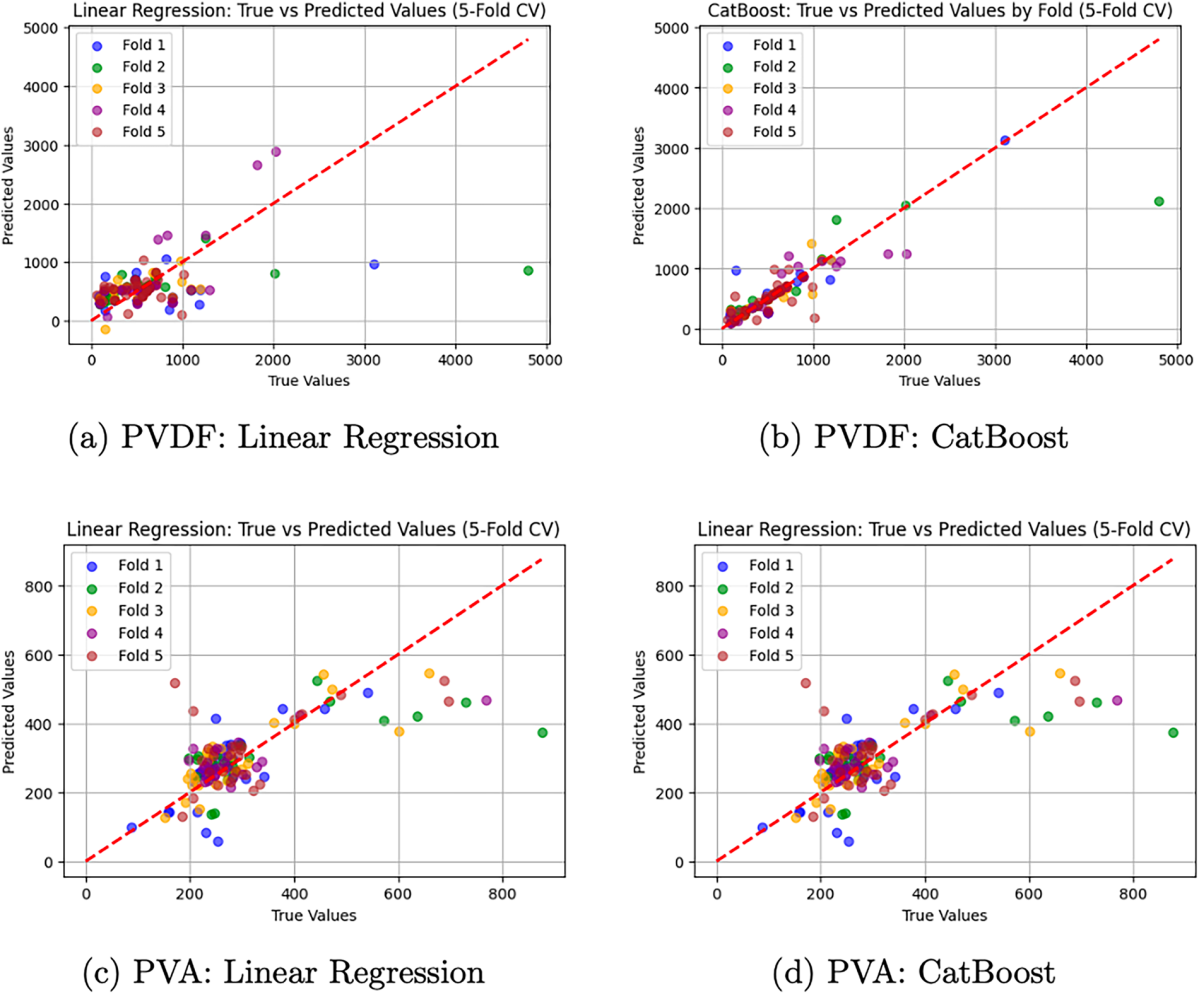
Comparison of predicted versus true fiber diameters using Linear Regression (left) and CatBoost (right) models for PVDF (top) and PVA (bottom) datasets. The dashed red line represents the *y* = *x* line. For PVDF, CatBoost performance metrics are *R*^2^ = 0.70 and MAE = 94 nm, compared to Linear Regression (*R*^2^ = 0.12 and MAE = 266 nm). For PVA, CatBoost metrics are *R*^2^ = 0.43 and MAE = 41 nm, compared to Linear Regression (*R*^2^ = 0.43 and MAE = 62 nm).

These results indicate that the dataset contains both strongly non-linear relationships and complex patterns, which can be captured by advanced machine learning models. The predictive performance observed across the two polymer systems demonstrates the dataset’s potential utility for machine learning applications in predicting fiber morphology from experimental parameters. The alignment of predicted and true values for both modeling approaches is illustrated in the corresponding scatter plots (Fig. 3).

For reproducibility, the models were configured as follows:

- **Linear Regression** was trained with the default settings of the standard library implementation.
- **CatBoost Regressor** was trained with the following hyperparameters:
- *iterations*: 500,
- *learning rate*: 0.05,
- *tree depth*: 6.
- *random seed*: 42.

## Usage Notes

The main objective of collecting and sharing the dataset is to propose a first dataset for 1) straightforward evaluation, visualization and analysis of electrospinning process parameters’ effect on nanofiber product morphology, 2) to accelerate research in data-driven electrospinning control. Moreover, the dataset intends to provide a foundation for machine learning modeling for electrospinning parameter prediction for a variety of materials and experimental configurations, which can be highly useful in the material exploration studies common to electrospinning, particularly in biomedical applications.

In particular, the following use-cases are naturally aligned with Cogni-e-Spin DB 1.0:

- **Process-structure-property analysis:** Researchers can systematically explore how processing parameters, material compositions, and environmental factors influence fiber morphology and diameter. This insight is crucial for optimizing electrospinning conditions tailored to applications such as biomedical scaffolds, filtration systems, and energy devices.
- **Data-driven modeling and machine learning:** The standardized and machine-readable dataset supports computational approaches, including supervised learning methods (e.g., regression models and neural networks for estimating quantitative metrics like average fiber diameter, or classification for identifying morphology types such as stable vs. unstable fiber formation). The dataset also enables advanced techniques like Anomaly detection (for detecting outliers or unusual diameter observations) and Explainable AI (XAI), such as SHAP or Feature Importance analysis, to determine which process, solution, or environmental parameters most influence fiber morphology.
- **Experimental design and optimization:** Predictive models trained on the dataset allow users to specify desired fiber characteristics and identify the corresponding process parameters, thereby accelerating experimental planning and reducing trial-and-error.

Further development of the dataset could be achieved through 1) literature screenings and 2) standardized data contributions from electrospinning research groups worldwide. Both types of data are highly sought after in the future to expand the dataset and make it even larger and more diverse for electrospinning experimental parameter prediction purposes. For 2), we have developed a companion web platform (https://electrospinning-data.org)^89^ that hosts a live version of the dataset. This platform allows researchers to directly contribute new electrospinning experimental records, as well as query them through a standardized interface. The live dataset can be used to source data for future versions of the Cogni-e-Spin DB dataset, following moderation and cleanup. To ensure the integrity and quality of community contributions, the platform employs a rigorous process for data validation, moderation, and versioning. This dual-validation workflow consists of two levels: an automated server check that enforces the completeness of fields and ensures all units are standardized for consistency across records, and a manual moderation step performed by an expert, who reviews entries for quality aspects requiring human judgment, such as checking for validity within expected ranges, placeholder values, and confirmation that only peer-reviewed published records are included. Successfully moderated data is appended to the live dataset on the web platform, ensuring only high-quality data is published publicly. The validated records from the live platform will then be compiled and formally released as future, versioned archives (e.g., Cogni-e-Spin DB 2.0) on permanent repositories (Zenodo), accompanied by a subsequent data descriptor to maintain long-term stability and reproducibility.

## Data Availability

The dataset is available on Zenodo at: 10.5281/zenodo.16731638. The preliminary dataset and the full list of sources are available on the TalTech Data Repository at 10.48726/vv924-p9b06.

## Code availability

The code used in this study is available at: https://github.com/taltechloc/Cogni-e-SpinDB. The source code for the electrospinning-data.org website is available at: https://github.com/taltechloc/electrospinning-data.org.
